# Supplementary material for: Therapeutic Effects of Morinda citrifolia Linn. (Noni) Aqueous Fruit Extract on the Glucose and Lipid Metabolism in High-Fat/High-Fructose-Fed Swiss Mice
Source: Nutrients. 2020 Nov 10;12(11):3439. doi: 10.3390/nu12113439 (PMC7696076; doi:10.3390/nu12113439)
Supplement: Supplementary file 1 [file nutrients-12-03439-s001.pdf]

Supplementary Materials

Table S1. Initial and final body weight, weight gain, food and calories intake and feed efficiency index between the 1st and 9th weeks.

Parameter.	Groups	
	CT (*n*=11)	HFF (*n*=31)
Initial Body Weight (g)	45.27±1.84	41.55±0.89
Final Body Weight (g)	47.91±1.75	54.97±1.25*
Weight Gain (g)	2.64±0.88	13.42±1.00*
Food intake (g/day)	45.36±1.71	27.89±0.28*
Calories intake (kcal)	172.4±6.49	149.1±1.70*
Feed Efficiency Index	47.80±1.75	54.80±1.25*

CT: regular chow diet. HFF: high-fat-high-fructose diet. Results are expressed as mean ± SEM. Student´s unpaired t-test. * = $p \leq 0.05$ vs. CT.

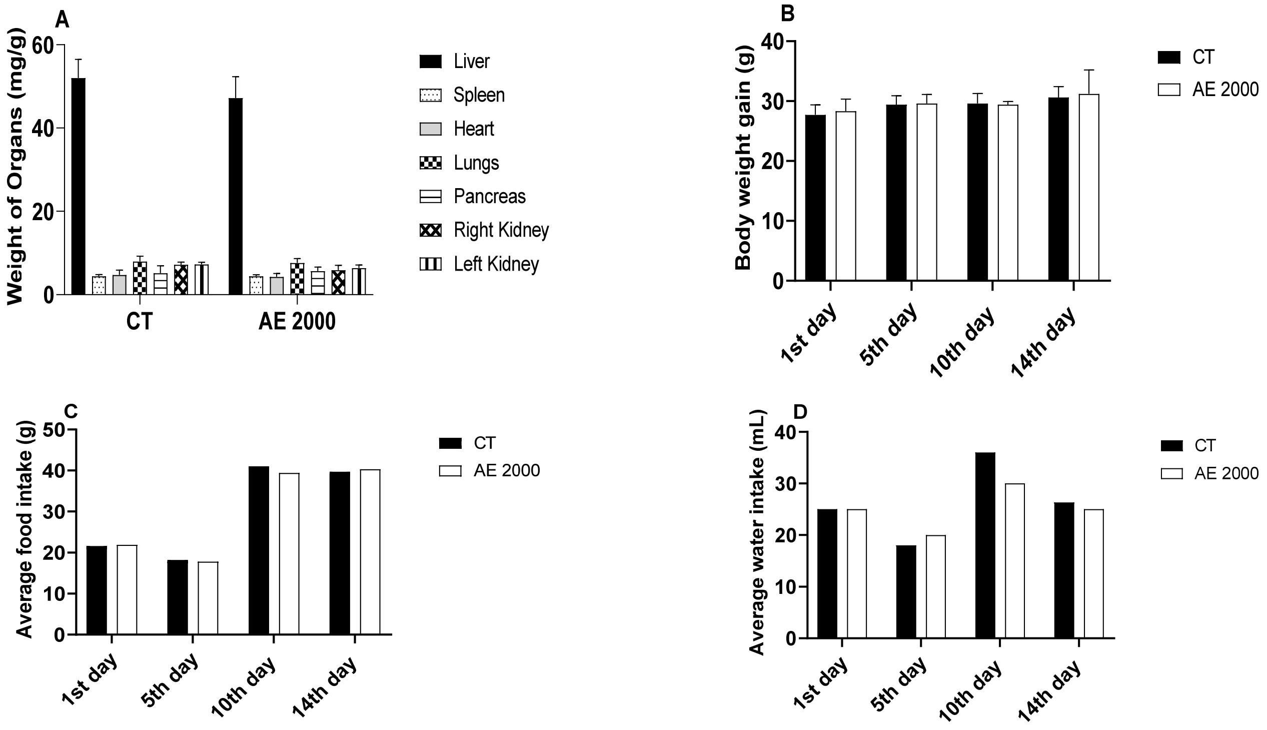

Figure S1. (A) Weight of organs of CT group and *M. citrifolia* aqueous fruit extract (AE) 2000 mg/kg after 14 days of acute oral toxicity test ; (B) Body weight gain of CT group and *M. citrifolia* aqueous fruit extract (AE) 2000 mg/kg during 14 days of acute oral toxicity test (1st, 5th, 10th and 14th days); (C) Average food intake of CT group and *M. citrifolia* aqueous fruit extract (AE) 2000 mg/kg during 14 days of acute oral toxicity test (1st, 5th, 10th and 14th days); (D) Average water intake of CT group and *M. citrifolia* aqueous fruit extract (AE) 2000 mg/kg during 14 days of acute oral toxicity test (1st, 5th, 10th and 14th days) of CT (regular chow diet + drink water, *n* = 5) and AE 2000 (regular chow diet + AE 2000 mg/kg, *n* = 5). Results are expressed as mean ± SEM. Two-way ANOVA followed by the Bonferroni post-test for weight of organs and body weight gain and Student´s unpaired t-test for average of food and water intake.

HIPPOCRATIC SCREENING TEST

Drug: *Morinda citrifolia* aqueous fruit extract **Dose:** 2000 mg/kg (oral gavage)
Time of gavage: 6:37 AM Data: 27/10/2017 **Animal:** female Swiss mice number 1 **Weight:** 27.5 g

**Symptoms**	**Normal**	**Time**											
		0	30 m	1,0 h	2,0 h	3,0 h	4,0 h	6,0 h	12 h	24 h	2 days	3 days	4 days
Normal appearance	4	4	4	4	4	4	4	4	4	4	4	4	4
Vocal phrenic	0	0	0	0	0	0	0	0	0	0	0	0	0
Irritability	0	0	0	0	0	0	0	0	0	0	0	0	0
Touch response	4	4	4	4	4	4	4	4	4	4	4	4	4
Tail grip	4	4	4	4	4	4	4	4	4	4	4	4	4
Contortion	0	0	0	0	0	0	0	0	0	0	0	0	0
Muscle tonus	4	4	4	4	4	4	4	4	4	4	4	4	4
Grip strength	4	4	4	4	4	4	4	4	4	4	4	4	4
Ataxia	0	0	0	0	0	0	0	0	0	0	0	0	0
Tremors	0	0	0	0	0	0	0	0	0	0	0	0	0
Convulsions	0	0	0	0	0	0	0	0	0	0	0	0	0
Estimulations	4	4	4	4	4	4	4	4	4	4	4	4	4
Hypnosis	0	0	0	0	0	0	0	0	0	0	0	0	0
Anesthesia	0	0	0	0	0	0	0	0	0	0	0	0	0
Lacrimation	0	0	0	0	0	0	0	0	0	0	0	0	0
Urination	4	4	4	4	4	4	4	4	4	4	4	4	4
Defecation	4	4	4	4	4	4	4	4	4	4	4	4	4
Hypothermia	0	0	0	0	0	0	0	0	0	0	0	0	0
Respiration	4	4	4	4	4	4	4	4	4	4	4	4	4
Cyanosis	0	0	0	0	0	0	0	0	0	0	0	0	0
Death	-	-	-	-	-	-	-	-	-	-	-	-	-

- **Codes:**
Tests with normal annotation "0", the intensity of the effect varies on a scale from 1 to 4
Test with normal annotation "4", the intensity of the effect can vary from 0 to 3 when there is a decrease, 4 when equal to the control and from 5 to 8 when there is an increase

**Symptoms**	**Normal**	5 days	6 days	7 days	8 days	9 days	10 days	11 days	12 days	13 days	14 days
Normal appearance	4	4	4	4	4	4	4	4	4	4	4
Vocal phrenic	0	0	0	0	0	0	0	0	0	0	0
Irritability	0	0	0	0	0	0	0	0	0	0	0
Touch response	4	4	4	4	4	4	4	4	4	4	4
Tail grip	4	4	4	4	4	4	4	4	4	4	4
Contortion	0	0	0	0	0	0	0	0	0	0	0
Muscle tonus	4	4	4	4	4	4	4	4	4	4	4
Grip strength	4	4	4	4	4	4	4	4	4	4	4
Ataxia	0	0	0	0	0	0	0	0	0	0	0
Tremors	0	0	0	0	0	0	0	0	0	0	0
Convulsions	0	0	0	0	0	0	0	0	0	0	0
Estimulations	4	4	4	4	4	4	4	4	4	4	4
Hypnosis	0	0	0	0	0	0	0	0	0	0	0
Anesthesia	0	0	0	0	0	0	0	0	0	0	0
Lacrimation	0	0	0	0	0	0	0	0	0	0	0
Urination	4	4	4	4	4	4	4	4	4	4	4
Defecation	4	4	4	4	4	4	4	4	4	4	4
Hypothermia	0	0	0	0	0	0	0	0	0	0	0
Respiration	4	4	4	4	4	4	4	4	4	4	4
Cyanosis	0	0	0	0	0	0	0	0	0	0	0
Death	-	-	-	-	-	-	-	-	-	-	-

HIPPOCRATIC SCREENING TEST

Drug: *Morinda citrifolia* aqueous fruit extract **Dose:** 2000 mg/kg (oral gavage)
Time of gavage: 6:41 AM Data: 27/10/2017 **Animal:** female Swiss mice
number 2 **Weight:** 30.4 g

Symptoms	Normal	Time											
		0	30 m	1,0 h	2,0 h	3,0 h	4,0 h	6,0 h	12 h	24 h	2 days	3 days	4 days
Normal appearance	4	4	4	4	4	4	4	4	4	4	4	4	4
Vocal phrenic	0	0	0	0	0	0	0	0	0	0	0	0	0
Irritability	0	0	0	0	0	0	0	0	0	0	0	0	0
Touch response	4	4	4	4	4	4	4	4	4	4	4	4	4
Tail grip	4	4	4	4	4	4	4	4	4	4	4	4	4
Contortion	0	0	0	0	0	0	0	0	0	0	0	0	0
Muscle tonus	4	4	4	4	4	4	4	4	4	4	4	4	4
Grip strength	4	4	4	4	4	4	4	4	4	4	4	4	4
Ataxia	0	0	0	0	0	0	0	0	0	0	0	0	0
Tremors	0	0	0	0	0	0	0	0	0	0	0	0	0
Convulsions	0	0	0	0	0	0	0	0	0	0	0	0	0
Estimulations	4	4	4	4	4	4	4	4	4	4	4	4	4
Hypnosis	0	0	0	0	0	0	0	0	0	0	0	0	0
Anesthesia	0	0	0	0	0	0	0	0	0	0	0	0	0
Lacrimation	0	0	0	0	0	0	0	0	0	0	0	0	0
Urination	4	4	4	4	4	4	4	4	4	4	4	4	4
Defecation	4	4	4	4	4	4	4	4	4	4	4	4	4
Hypothermia	0	0	0	0	0	0	0	0	0	0	0	0	0
Respiration	4	4	4	4	4	4	4	4	4	4	4	4	4
Cyanosis	0	0	0	0	0	0	0	0	0	0	0	0	0
Death	-	-	-	-	-	-	-	-	-	-	-	-	-

- **Codes:**
Tests with normal annotation "0", the intensity of the effect varies on a scale from 1 to 4
Test with normal annotation "4", the intensity of the effect can vary from 0 to 3 when there is a decrease, 4 when equal to the control and from 5 to 8 when there is an increase

Symptoms	Normal	5 days	6 days	7 days	8 days	9 days	10 days	11 days	12 days	13 days	14 days
Normal appearance	4	4	4	4	4	4	4	4	4	4	4
Vocal phrenic	0	0	0	0	0	0	0	0	0	0	0
Irritability	0	0	0	0	0	0	0	0	0	0	0
Touch response	4	4	4	4	4	4	4	4	4	4	4
Tail grip	4	4	4	4	4	4	4	4	4	4	4
Contortion	0	0	0	0	0	0	0	0	0	0	0
Muscle tonus	4	4	4	4	4	4	4	4	4	4	4
Grip strength	4	4	4	4	4	4	4	4	4	4	4
Ataxia	0	0	0	0	0	0	0	0	0	0	0
Tremors	0	0	0	0	0	0	0	0	0	0	0
Convulsions	0	0	0	0	0	0	0	0	0	0	0
Estimulations	4	4	4	4	4	4	4	4	4	4	4
Hypnosis	0	0	0	0	0	0	0	0	0	0	0
Anesthesia	0	0	0	0	0	0	0	0	0	0	0
Lacrimation	0	0	0	0	0	0	0	0	0	0	0
Urination	4	4	4	4	4	4	4	4	4	4	4
Defecation	4	4	4	4	4	4	4	4	4	4	4
Hypothermia	0	0	0	0	0	0	0	0	0	0	0
Respiration	4	4	4	4	4	4	4	4	4	4	4
Cyanosia	0	0	0	0	0	0	0	0	0	0	0
Death	-	-	-	-	-	-	-	-	-	-	-

HIPPOCRATIC SCREENING TEST

Drug: *Morinda citrifolia* aqueous fruit extract **Dose:** 2000 mg/kg (oral gavage)
Time of gavage: 6:44 AM Data: 27/10/2017 **Animal:** female Swiss mice number 3 **Weight:** 29.1 g

Symptoms	Normal	Time												
		0	30 m	1,0 h	2,0 h	3,0 h	4,0 h	6,0 h	12 h	24 h	2 days	3 days	4 days
Normal appearance	4	4	4	4	4	4	4	4	4	4	4	4	4
Vocal phrenic	0	0	0	0	0	0	0	0	0	0	0	0	0
Irritability	0	0	0	0	0	0	0	0	0	0	0	0	0
Touch response	4	4	4	4	4	4	4	4	4	4	4	4	4
Tail grip	4	4	4	4	4	4	4	4	4	4	4	4	4
Contortion	0	0	0	0	0	0	0	0	0	0	0	0	0
Muscle tonus	4	4	4	4	4	4	4	4	4	4	4	4	4
Grip strength	4	4	4	4	4	4	4	4	4	4	4	4	4
Ataxia	0	0	0	0	0	0	0	0	0	0	0	0	0
Tremors	0	0	0	0	0	0	0	0	0	0	0	0	0
Convulsions	0	0	0	0	0	0	0	0	0	0	0	0	0
Estimulations	4	4	4	4	4	4	4	4	4	4	4	4	4
Hypnosis	0	0	0	0	0	0	0	0	0	0	0	0	0
Anesthesia	0	0	0	0	0	0	0	0	0	0	0	0	0
Lacrimation	0	0	0	0	0	0	0	0	0	0	0	0	0
Urination	4	4	4	4	4	4	4	4	4	4	4	4	4
Defecation	4	4	4	4	4	4	4	4	4	4	4	4	4
Hypothermia	0	0	0	0	0	0	0	0	0	0	0	0	0
Respiration	4	4	4	4	4	4	4	4	4	4	4	4	4
Cyanosis	0	0	0	0	0	0	0	0	0	0	0	0	0
Death	-	-	-	-	-	-	-	-	-	-	-	-	-

- **Codes:**
Tests with normal annotation "0", the intensity of the effect varies on a scale from 1 to 4
Test with normal annotation "4", the intensity of the effect can vary from 0 to 3 when there is a decrease, 4 when equal to the control and from 5 to 8 when there is an increase

Symptoms	Normal	5 days	6 days	7 days	8 days	9 days	10 days	11 days	12 days	13 days	14 days
Normal appearance	4	4	4	4	4	4	4	4	4	4	4
Vocal phrenic	0	0	0	0	0	0	0	0	0	0	0
Irritability	0	0	0	0	0	0	0	0	0	0	0
Touch response	4	4	4	4	4	4	4	4	4	4	4
Tail grip	4	4	4	4	4	4	4	4	4	4	4
Contortion	0	0	0	0	0	0	0	0	0	0	0
Muscle tonus	4	4	4	4	4	4	4	4	4	4	4
Grip strength	4	4	4	4	4	4	4	4	4	4	4
Ataxia	0	0	0	0	0	0	0	0	0	0	0
Tremors	0	0	0	0	0	0	0	0	0	0	0
Convulsions	0	0	0	0	0	0	0	0	0	0	0
Estimulations	4	4	4	4	4	4	4	4	4	4	4
Hypnosis	0	0	0	0	0	0	0	0	0	0	0
Anesthesia	0	0	0	0	0	0	0	0	0	0	0
Lacrimation	0	0	0	0	0	0	0	0	0	0	0
Urination	4	4	4	4	4	4	4	4	4	4	4
Defecation	4	4	4	4	4	4	4	4	4	4	4
Hypothermia	0	0	0	0	0	0	0	0	0	0	0
Respiration	4	4	4	4	4	4	4	4	4	4	4
Cyanosis	0	0	0	0	0	0	0	0	0	0	0
Death	-	-	-	-	-	-	-	-	-	-	-

HIPPOCRATIC SCREENING TEST

Drug: *Morinda citrifolia* aqueous fruit extract **Dose:** 2000 mg/kg (oral gavage)
Time of gavage: 6:49 AM Data: 27/10/2017 **Animal:** female Swiss mice
number 4 **Weight:** 25.2 g

Symptoms	Normal	Time											
		0	30 m	1,0 h	2,0 h	3,0 h	4,0 h	6,0 h	12 h	24 h	2 days	3 days	4 days
Normal appearance	4	4	4	4	4	4	4	4	4	4	4	4	4
Vocal phrenic	0	0	0	0	0	0	0	0	0	0	0	0	0
Irritability	0	0	0	0	0	0	0	0	0	0	0	0	0
Touch response	4	4	4	4	4	4	4	4	4	4	4	4	4
Tail grip	4	4	4	4	4	4	4	4	4	4	4	4	4
Contortion	0	0	0	0	0	0	0	0	0	0	0	0	0
Muscle tonus	4	4	4	4	4	4	4	4	4	4	4	4	4
Grip strength	4	4	4	4	4	4	4	4	4	4	4	4	4
Ataxia	0	0	0	0	0	0	0	0	0	0	0	0	0
Tremors	0	0	0	0	0	0	0	0	0	0	0	0	0
Convulsions	0	0	0	0	0	0	0	0	0	0	0	0	0
Estimulations	4	4	4	4	4	4	4	4	4	4	4	4	4
Hypnosis	0	0	0	0	0	0	0	0	0	0	0	0	0
Anesthesia	0	0	0	0	0	0	0	0	0	0	0	0	0
Lacrimation	0	0	0	0	0	0	0	0	0	0	0	0	0
Urination	4	4	4	4	4	4	4	4	4	4	4	4	4
Defecation	4	4	4	4	4	4	4	4	4	4	4	4	4
Hypothermia	0	0	0	0	0	0	0	0	0	0	0	0	0
Respiration	4	4	4	4	4	4	4	4	4	4	4	4	4
Cyanosis	0	0	0	0	0	0	0	0	0	0	0	0	0
Death	-	-	-	-	-	-	-	-	-	-	-	-	-

- **Codes:**

Tests with normal annotation "0", the intensity of the effect varies on a scale from 1 to 4
Test with normal annotation "4", the intensity of the effect can vary from 0 to 3 when there is a decrease, 4 when equal to the control and from 5 to 8 when there is an increase

Symptoms	Normal	5 days	6 days	7 days	8 days	9 days	10 days	11 days	12 days	13 days	14 days
Normal appearance	4	4	4	4	4	4	4	4	4	4	4
Vocal phrenic	0	0	0	0	0	0	0	0	0	0	0
Irritability	0	0	0	0	0	0	0	0	0	0	0
Touch response	4	4	4	4	4	4	4	4	4	4	4
Tail grip	4	4	4	4	4	4	4	4	4	4	4
Contortion	0	0	0	0	0	0	0	0	0	0	0
Muscle tonus	4	4	4	4	4	4	4	4	4	4	4
Grip strength	4	4	4	4	4	4	4	4	4	4	4
Ataxia	0	0	0	0	0	0	0	0	0	0	0
Tremors	0	0	0	0	0	0	0	0	0	0	0
Convulsions	0	0	0	0	0	0	0	0	0	0	0
Estimulations	4	4	4	4	4	4	4	4	4	4	4
Hypnosis	0	0	0	0	0	0	0	0	0	0	0
Anesthesia	0	0	0	0	0	0	0	0	0	0	0
Lacrimation	0	0	0	0	0	0	0	0	0	0	0
Urination	4	4	4	4	4	4	4	4	4	4	4
Defecation	4	4	4	4	4	4	4	4	4	4	4
Hypothermia	0	0	0	0	0	0	0	0	0	0	0
Respiration	4	4	4	4	4	4	4	4	4	4	4
Cyanosis	0	0	0	0	0	0	0	0	0	0	0
Death	-	-	-	-	-	-	-	-	-	-	-

HIPPOCRATIC SCREENING TEST

Drug: *Morinda citrifolia* aqueous fruit extract **Dose:** 2000 mg/kg (oral gavage)
Time of gavage: 6:53 AM PM Data: 27/10/2017 **Animal:** female Swiss mice number 5 **Weight:** 29.4 g

Symptoms	Normal	Time											
		0	30 m	1,0 h	2,0 h	3,0 h	4,0 h	6,0 h	12 h	24 h	2 days	3 days	4 days
Normal appearance	4	4	4	4	4	4	4	4	4	4	4	4	4
Vocal phrenic	0	0	0	0	0	0	0	0	0	0	0	0	0
Irritability	0	0	0	0	0	0	0	0	0	0	0	0	0
Touch response	4	4	4	4	4	4	4	4	4	4	4	4	4
Tail grip	4	4	4	4	4	4	4	4	4	4	4	4	4
Contortion	0	0	0	0	0	0	0	0	0	0	0	0	0
Muscle tonus	4	4	4	4	4	4	4	4	4	4	4	4	4
Grip strength	4	4	4	4	4	4	4	4	4	4	4	4	4
Ataxia	0	0	0	0	0	0	0	0	0	0	0	0	0
Tremors	0	0	0	0	0	0	0	0	0	0	0	0	0
Convulsions	0	0	0	0	0	0	0	0	0	0	0	0	0
Estimulations	4	4	4	4	4	4	4	4	4	4	4	4	4
Hypnosis	0	0	0	0	0	0	0	0	0	0	0	0	0
Anesthesia	0	0	0	0	0	0	0	0	0	0	0	0	0
Lacrimation	0	0	0	0	0	0	0	0	0	0	0	0	0
Urination	4	4	4	4	4	4	4	4	4	4	4	4	4
Defecation	4	4	4	4	4	4	4	4	4	4	4	4	4
Hypothermia	0	0	0	0	0	0	0	0	0	0	0	0	0
Respiration	4	4	4	4	4	4	4	4	4	4	4	4	4
Cyanosis	0	0	0	0	0	0	0	0	0	0	0	0	0
Death	-	-	-	-	-	-	-	-	-	-	-	-	-

- **Codes:**
Tests with normal annotation "0", the intensity of the effect varies on a scale from 1 to 4
Test with normal annotation "4", the intensity of the effect can vary from 0 to 3 when there is a decrease, 4 when equal to the control and from 5 to 8 when there is an increase

Symptoms	Normal	5 days	6 days	7 days	8 days	9 days	10 days	11 days	12 days	13 days	14 days
Normal appearance	4	4	4	4	4	4	4	4	4	4	4
Vocal phrenic	0	0	0	0	0	0	0	0	0	0	0
Irritability	0	0	0	0	0	0	0	0	0	0	0
Touch response	4	4	4	4	4	4	4	4	4	4	4
Tail grip	4	4	4	4	4	4	4	4	4	4	4
Contortion	0	0	0	0	0	0	0	0	0	0	0
Muscle tonus	4	4	4	4	4	4	4	4	4	4	4
Grip strength	4	4	4	4	4	4	4	4	4	4	4
Ataxia	0	0	0	0	0	0	0	0	0	0	0
Tremors	0	0	0	0	0	0	0	0	0	0	0
Convulsions	0	0	0	0	0	0	0	0	0	0	0
Estimulations	4	4	4	4	4	4	4	4	4	4	4
Hypnosis	0	0	0	0	0	0	0	0	0	0	0
Anesthesia	0	0	0	0	0	0	0	0	0	0	0
Lacrimation	0	0	0	0	0	0	0	0	0	0	0
Urination	4	4	4	4	4	4	4	4	4	4	4
Defecation	4	4	4	4	4	4	4	4	4	4	4
Hypothermia	0	0	0	0	0	0	0	0	0	0	0
Respiration	4	4	4	4	4	4	4	4	4	4	4
Cyanosia	0	0	0	0	0	0	0	0	0	0	0
Death	-	-	-	-	-	-	-	-	-	-	-

Figure S2. Hippocratic Screening Test of AE 2000 mg/kg group (n = 5) during 14 days of acute oral toxicity test.

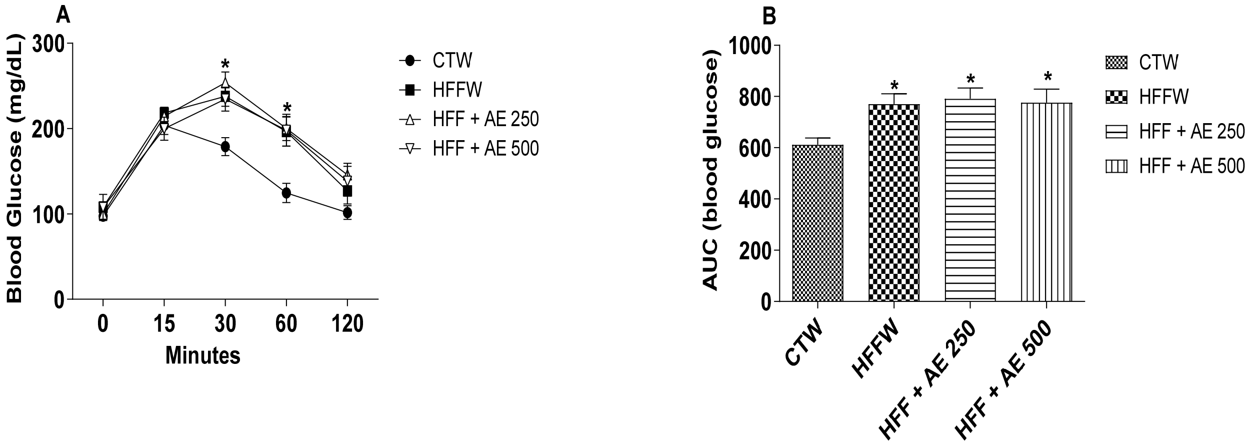

Figure S3. Evaluation of the glycemic profile at the beginning of the treatment with AE. (A) Oral glucose tolerance test at the end of the treatment (9th week); (B) Area under the curve (AUC) of blood glucose of animals evaluated at the end of the treatment (9th week) of CTW (CT + drink water, *n* = 11); HFFW (high-fat-high-fructose diet + drink water, *n* = 11); HFF + AE 250 (HFF + *M. citrifolia* fruit aqueous extract of 250 mg/kg of body weight, *n* = 11). HFF + AE 500 (HFF + *M. citrifolia* fruit aqueous extract of 500 mg/kg, *n* = 10) groups. Results are expressed as mean ± SEM. * = $p \leq 0.05$ vs. CTW. Two-way ANOVA followed by Bonferroni post-test for oral glucose tolerance test. ANOVA followed by Tukey post-test for AUC.
